# Freeze-Dried Mesenchymal Stem Cell-Secretome Pharmaceuticalization: Optimization of Formulation and Manufacturing Process Robustness

**DOI:** 10.3390/pharmaceutics13081129

**Published:** 2021-07-23

**Authors:** Michela Mocchi, Elia Bari, Giorgio Marrubini, Andrea Foglio Bonda, Sara Perteghella, Fulvio Tartara, Fabio Cofano, Giuseppe di Perna, Lorella Giovannelli, Delia Mandracchia, Marzio Sorlini, Diego Garbossa, Maria Luisa Torre, Lorena Segale

**Affiliations:** 1 Department of Drug Sciences, University of Pavia, Viale Taramelli 12, I-27100 Pavia, Italy; michela.mocchi@unipv.it (M.M.); elia.bari@unipv.it (E.B.); giorgio.marrubini@unipv.it (G.M.); sara.perteghella@unipv.it (S.P.); 2 Department of Pharmaceutical Sciences, University of Piemonte Orientale, Largo Donegani 2/3, I-28100 Novara, Italy; andrea.fogliobonda@uniupo.it (A.F.B.); lorella.giovannelli@uniupo.it (L.G.); lorena.segale@uniupo.it (L.S.); 3 APTsol S.r.l., Largo Guido Donegani 2/3, I-28100 Novara, Italy; 4 PharmaExceed S.r.l., Piazza Castello 19, I-27100 Pavia, Italy; marzio.sorlini@supsi.ch; 5 Fondazione IRCCS Istituto Neurologico Nazionale Mondino, Via Mondino 2, I-27100 Pavia, Italy; tartarafulvio@gmail.com; 6 Neuroscience Department “Rita Levi Montalcini” Via Cherasco 15, I-10126 Torino, Italy; fabio.cofano@gmail.com (F.C.); dr.giuseppediperna@gmail.com (G.d.P.); dgarbossa@gmail.com (D.G.); 7 Vertebral Surgery Unit, Humanitas Gradenigo, Corso Regina Margherita 8, I-10153 Turin, Italy; 8 Department of Molecular and Translational Medicine, University of Brescia, Viale Europa 11, I-25123 Brescia, Italy; delia.mandracchia@unibs.it; 9 SUPSI-Department of Innovative Technologies, Lugano University Centre, Campus Est, Via la Santa 1, CH-6962 Viganello, Switzerland

**Keywords:** mesenchymal stem cells, secretome, freeze-drying, formulation

## Abstract

Producing mesenchymal stem cell (MSC)-secretome for dose escalation studies and clinical practice requires scalable and good manufacturing practice (GMP)-compliant production procedures and formulation into a standardized medicinal product. Starting from a method that combines ultrafiltration and freeze-drying to transform MSC-secretome into a pharmaceutical product, the lyosecretome, this work aims to: (i) optimize the lyosecretome formulation; (ii) investigate sources of variability that can affect the robustness of the manufacturing process; (iii) modify the ultrafiltration step to obtain a more standardized final product. Design of experiments and principal component analysis of the data were used to study the influence of batch production, lyophilization, mannitol (M)/sucrose (S) binary mixture, selected as cryoprotectant excipients, and the total amount of excipients on the extracellular vesicles (EV) particle size, the protein and lipid content and the in vitro anti-elastase. The different excipients ratios did not affect residual moisture or EV particle size; simultaneously, proteins and lipids were better preserved in the freeze-dried product using the maximum total concentration of excipients (1.5% *w*/*v*) with a M:S ratio of about 60% *w*/*w*. The anti-elastase activity was instead better preserved using 0.5% *w*/*w* of M as excipient. The secretome batch showed to be the primary source of variability; therefore, the manufacturing process has been modified and then validated: the final product is now concentrated to reach a specific protein (and lipid) concentration instead of cell equivalent concentration. The new standardization approach led to a final product with more reproducible quali-quantitative composition and higher biological activity.

## 1. Introduction

In the 1990s, the multipotency of mesenchymal stem cells (MSCs) was identified as a promise for developing new and more effective cell therapies intended to revolutionize the clinical practice in regenerative medicine and improve the patient quality of life [1,2]. Since then, more than 800 worldwide clinical trials have proven MSC therapeutic effectiveness and safety, spanning from cardiovascular to neurological, from tegumentary to respiratory (source: http://clinicaltrials.gov, term search: Mesenchymal Stem Cells, last search 18 May 2021). Twenty years later from their discovery, Caplan suggested renaming MSCs as “Medicinal Signalling Cells” since multipotency seemed to be no longer the key aspect of their therapeutic effects [3]. In fact, after transplantation, the number of MSCs that effectively engraft and differentiate into the damaged tissue is very low, suggesting a paracrine mechanism of action [4,5,6].

The substances secreted by MSCs that modulate the resident cell responses are collectively named secretome, composed of free-soluble factors (including cytokines, chemokines and growth factors) and insoluble nano/microstructured extracellular vesicles (EVs) [7,8]. MSC-secretome can reproduce the therapeutic effects of stem cells themselves and cell-free therapies should provide numerous advantages compared with whole-cell MSC infusions in terms of safety and technological advantages [9]. Unfortunately, it remains a significant challenge translating this therapy into the clinic: with the focus on therapeutic applications, conventional manufacturing processes (such as ultracentrifugation or chromatography) limit secretome applications and the ability to evaluate safety and efficacy at high doses on a large animal or clinical trials [10,11]. Producing MSC-secretome for dose escalation studies and clinical practice requires a scalable production procedure, including raw materials or consumables, compatible with current good manufacturing practice (GMP) procedures [12]. Finally, to have a meaningful role in medicine, the secretome needs to be turned into a format easy to manage by the clinical community. In other words, MSC-secretome needs, as for all the Active Pharmaceutical Ingredients, to be formulated into a standardized medicinal product [7]. In this regard, recently, Bari and co-workers proposed a method to transform MSC-secretome into a pharmaceutical product for its large-scale production [13]. For this purpose, ultrafiltration and freeze-drying were combined: MSC-secretome was purified from culture supernatants by ultrafiltration in a GMP-compliant cell factory, concentrated at 0.5 × 10^6^ cell equivalents per mL, added with cryoprotectant and lyophilized. A freeze-dried and “ready-off-the-shelf” powder–the lyosecretome–containing extracellular vesicles and proteins was obtained. Although the described process is successful in scalability and GMP compliance, it fails to obtain a product standardized in protein and lipid content and thus in vitro biological activity. Such batch-to-batch variability has also been reported in terms of biological activity for secretomes derived from both humans [14] or equine MSCs [15]. Indeed, like any other biological drug, the starting point for the production of the secretome is a living organism, the cell and the process mentioned above standardizes the final product in terms of cell equivalents: a precise quantity of the final product is obtained by a specific number of cells. Therefore, the amount of proteins and lipids of the final product depends on how many of them have been produced by that number of cells. With this in mind, it is questionable whether some process variables can be modified to optimize the manufacturing process. Moreover, to protect biological material, especially EVs, mannitol was chosen as a stabilizer from freeze-drying stresses. The tendency of mannitol to maintain its crystalline form is well appreciated for giving the best cake appearance and a high collapse temperature, associated with a shorter freeze-drying time [16]. However, other stabilizers may be used to avoid affecting secretome components in quality and quantity, such as other sugars/polyols, proteins and amino acids or surfactants [17]. Especially, sucrose is appreciated for its vitrification capability: forming an amorphous phase with high viscosity it may help to protect the lipid layer of the EVs and to reduce the merge of vesicles [18].

Given these premises, this work aims to: (i) optimize the lyosecretome formulation; (ii) investigate sources of variability that can affect the robustness of the manufacturing process described by Bari and colleagues [13]; (iii) modify the concentration step to obtain a more standardized final product. At first, a multi-level 5 × 2 factorial design was used to optimize lyosecretome formulation, using mannitol and sucrose as excipients. Then, a quadratic D-optimal design was computed to evaluate the effect of secretome batch (S), lyophilization (L), concentration of excipients (C) and the mannitol/sucrose ratio in the excipient mixture (M, to indicate the proportion of mannitol in the binary mixture mannitol/sucrose) on the pharmaceutical dosage form characteristics (residual humidity and cake appearance) and on the product performances (protein and lipid yields, the EV particle size and in vitro anti-elastase activity). Finally, the manufacturing process has been modified to reduce batch-to-batch variability: the final product is now concentrated to reach a specific protein (and lipid) concentration instead of cell equivalent concentration. The ability of the new standardization method in obtaining a final product with more reproducible quali-quantitative composition and biological activity has been investigated and the modified manufacturing process has been validated.

## 2. Materials and Methods

### 2.1. Materials

Reagents used for the cell culture were purchased from Euroclone (Milan, Italy) and platelet lysate (PLy) from Sclavo Diagnostics (Siena, Italy). Acetone, bovine serum albumin (BSA), collagenase, mannitol, Nile red, phosphatidylcholine and sucrose were obtained from Sigma–Aldrich (Milan, Italy). Epigallocatechin gallate (EGCG), *N*-succinyl-Ala-Ala-Ala-p-nitroanilide and pancreatic porcine elastase were from Merck Life Science S.r.l., Milan, Italy.

### 2.2. Design of Experiments

A multi-level 5 × 2 design was used to understand how formulation attributes can influence product performances (Table 1). In detail, a mannitol/sucrose binary mixture was selected as an excipient. Variable 1 (coded V1) represented the percentage of mannitol in the mixture and had 5 levels corresponding to the 25, 45, 65, 85 and 100% *w*/*v* (coded as −1.0, −0.5, 0.0, 0.6, 1.0). Variable 2 (V2) represented the amount of the mixture expressed as % *w*/*v* in the pre-freeze-dryer solutions. For V2, two levels (−1 and +1) were selected, corresponding to 0.5 and 1.5% *w*/*v*.

### 2.3. Sample Preparation

#### 2.3.1. Isolation and Expansion of Human Adipose-Derived MSCs (AD-MSCs)

AD-MSCs were harvested from adipose tissues collected from patients undergoing abdominoplasty, as previously reported [19,20], after informed consent (ASST Grande Ospedale Metropolitano Niguarda, Milan, Ref. 12 November 2009). MSCs were seeded into flasks (10,000 cells/cm^2^) at 37 °C and 5% CO_2_ and cultured in complete culture medium (DMEM/F12 minimal medium plus 5% *v*/*v* PLy, plus 1% *v*/*v* penicillin/streptomycin and 1% *v*/*v* amphotericin B) until passage 3. Then, secretome release was induced by culturing MSCs in DMEM/F12 without platelet lysate for 48 h. MSCs were characterized to assess their identity according to the International Society for Cellular Therapy [21].

#### 2.3.2. MSC-Secretome Ultrafiltration

Conditioned media was centrifuged at 3500× *g* for 10 min to eliminate cell fragments and apoptotic bodies. Afterwards, supernatants were collected and the MSC-secretome purification process was performed by tangential flow filtration using the KrosFlo^®^ Research 2i system (Spectrum Laboratories, Milan, Italy) using a 5 kDa Molecular Weight Cut Off (MWCO) filtration module (Spectrum Laboratories, Milan, Italy). Before proceeding with the ultrafiltration, all the instrument units were sterilized to operate in aseptic conditions under a laminar flow hood in a B cleanroom suite. The automated process allowed, at first, to concentrate and then to diafilter the samples. According to the manufacturer’s guidelines, during each step, the shear rate of the feed stream was kept between 2000 s^−1^ and 6000 s^−1^, while the trans-membrane pressure index did not exceed 5 psi. In the first part of the manuscript, the concentration process ended when a concentration of 0.5 × 10^6^ cell equivalents per mL was achieved (CE). In the second part, the concentration process ended when a concentration of 115 µg/mL of proteins was reached (PL). Sterilized and ultrapure water was used as a dialysis buffer. 

#### 2.3.3. Freeze-Drying

Under Bio-Hood (Steril-VBH EuroClone S.p.a., Milan, Italy), vials (Colaver Srl, Vimodrone, Italy) were filled with 1 mL of solution using a manual pipette. Stoppers were pre-labelled to differentiate formulations and vials containing the different formulations were randomly placed on the bottomless shelves. Four temperature probes were installed in defined vials. The freeze dryer (Epsilon 2-6D LSCplus, Martin Christ GmbH, Osterode am Harz, Germany) was loaded, protecting the drying chamber from dust with a laminar air flow cabinet equipped with a fan filtration unit (Success Way Clean Technology Co. Ltd., Suzhou, Jiangsu, China) and the freeze-dryer polyacrylate panel was covered with an isolation panel built on our own. A conservative freeze-drying process was adopted (parameters are reported in Table 2) and the well-known “freezing step program” was applied to reduce samples variability. At the end of the process, vacuum stoppering was carried out, the freeze-dryer was unloaded and vials were crimped and labelled. Vials were stored at −20 °C until use (maximum six months).

### 2.4. Sample Characterization 

#### 2.4.1. Residual Moisture

The test was carried out with Coulometric Titrator HI904 (Hanna Instruments, Villafranca Padovana, Italy). An external extraction method with an anolyte solution was adopted. In detail, approximately 2.5 mL of anolyte (Hydranal^TM^ Culomat AD, Honeywell, Charlotte, NC, USA) were injected into the septum bottle by a syringe. The weight of the anolyte injected was determined by back weighing. The vial was sonicated for 30 min at 40 °C and then an aliquot of the dispersion/solution was drawn again into the same syringe, weighed and injected into the titration cell. Again, the weight was determined by back weighing. Titration was performed twice for each vial. The vial back pressure inlet was controlled with a syringe containing molecular sieves to reduce ambient moisture influence. All the samples were allowed to equilibrate at ambient temperature before analyses.

#### 2.4.2. Cake Aspect

Four vials for each formulation were randomly removed from −20 °C and equilibrated at room temperature for 24 h before performing the cake aspect evaluation. A cake aspect questionnaire was submitted to five of the authors having experience in freeze-drying (M.M., E.B., A.F.B, L.G., L.S). The subject shall give a score to 6 selected typical defects observed in the freeze-dried cake. The following defects were evaluated: (i) collapse, (ii) cake shrinkage, (iii) cracked cake, (iv) skin formation, (v) minor splashing and (vi) non-uniform cake appearance. The subjects evaluated the appearance of the cakes giving a score from 0 to 5, where five corresponded to a higher level of noticeable defect. A visual legend (adapted from Patel et al. [22] and showing images of the defects) was provided to the subjects to reduce individual variability. For each defect, the score was calculated by summing the five single evaluators’ outcomes and removing the corresponding minimum and maximum expressed values.

#### 2.4.3. Total Protein Content

A micro BCA-Protein Assay Kit (Thermo Fischer Scientific, Milan, Italy) was used following the manufacturer’s instructions to evaluate the total protein content of the samples before and after lyophilization. Freeze-dried samples were first resuspended in deionized water. The absorbance–concentration calibration curve was produced using bovine serum albumin (BSA) as standard. Working reagent solution was added to each sample and the calibration curve (ratio 1:1), then incubated at 37 °C for 2 h before reading. The absorbance was measured at 562 nm using a microplate reader (Synergy HT, Milan, Italy). The concentration of unknown protein content was calculated from a plot of concentration vs. absorbance obtained for the standard protein solutions, using a third-order polynomial equation, with R^2^ = 0.99. Each sample was measured in triplicate.

#### 2.4.4. Phospholipid Quantification by Nile Red Assay

A Nile Red stock solution was prepared starting from Nile Red powder dissolved in acetone (3.14 M). The stock solution was stored at 4 °C in the dark, avoiding light exposure. The stock solution was diluted 100× using filtered PBS (pH = 7.14) and 10 µL of it were incubated with 90 µL of samples. After 5 min, Synergy HT measured the relative fluorescence at fixed wavelengths (530/25 excitation and 645/40 emission). The fluorescence–concentration calibration curve was developed using phosphatidylcholine (PC) as standard, with R^2^ = 0.99. Each sample was tested in triplicate.

#### 2.4.5. EV Particle Size Determination

The freeze-dried samples were dispersed in 1 mL of deionized water and analyzed by Nanoparticle Tracking Analysis (NTA, NanoSight NS 300 equipment, Malvern Panalytical Ltd., Malvern, UK)). Measurements were carried out at room temperature with a detection angle of 90° and the NTA software elaborated the data. All analyses were in triplicate.

#### 2.4.6. Anti-Elastase Activity

The anti-elastase activity of all samples has been evaluated by an in vitro method, as previously reported [15]. Briefly, the substrate *N*-succinyl-Ala-Ala-Ala-p-nitroanilide was dissolved in TRIS buffer at 0.41 mmol/L and the pancreatic porcine elastase was solubilized in phosphate buffer pH 6.8 at 0.5 IU/mL. All the samples were incubated with the enzyme for 20 min. Then, the substrate was added and the kinetic reaction was monitored by spectrophotometric analysis (Synergy HT) at the absorbance of 410 nm for 60 min (one measurement each minute). EGCG was used as a positive control (at 7.2 mg/mL), while the reaction mixture in the absence of sample was used as a negative control. Analyses were performed in triplicate. 

### 2.5. D-Optimal Design

All calculations were performed using Microsoft Excel and R version 3.1.0 (2014-04-10) Copyright (C) 2014 The R Foundation for Statistical Computing. R-based chemometric software routines were used for the design of experiments calculations. The R-based software has been developed by the Group of Chemometrics of the Italian Chemical Society [23]. The protein and lipid yield results and the EV particle size (Y_P_, Y_L_ and Y_NTA_, respectively) were fitted by a D-optimal design computed to study the information collected and summarized in the section of the Supplementary Material (Tables S1 and S2). In detail, the study was conducted by evaluating the effect of four factors on the responses: the secretome batch (S), the lyophilization (L), the concentration of excipients (C) and the concentration ratio of mannitol and sucrose (M, to indicate the proportion of mannitol in the binary mixture mannitol:sucrose). Table 3 summarizes the factors and levels at which they were studied.

For proteins and lipids, two non-canonical quadratic models were postulated that included two qualitative factors (S and L, studied at three and two levels, respectively) and two continuous factors, C and M. The factor C was studied at two levels. In contrast, the M factor was examined at five different concentration levels with respect to sucrose (25/75, 45/55, 65/35, 85/15, 100/0). The postulated model for both responses is the following:

Y_K_ = b_0_ + b_1_ × S_1_ + b_2_ × S_2_ + b_2_ × L + b_3_ × C + b_4_ × M + b_44_ × M^2^ (1)

with K = P, L. The coding of the qualitative factor at three levels is S_1_ = 1 and S_2_ = 0 referring to the batch 1, S_1_ = 0, S_2_ = 1 to batch 2 and S_1_ = S_2_ = 0 to batch 3. 

For EV particle size, the postulated model is the following:

Y_NTA_ = b_0_ + b_1_ × S + b_2_ × C + b_3_ × M + b_33_ × M^2^ (2)

In this case, the qualitative factor lyophilization (L) was fixed because only lyophilized formulations were studied.

For in vitro anti-elastase activity, all the data reported in each row of Table S1 are strongly correlated with each other since they originate from enzymatic kinetics [24] and can thus be represented with a very good fit by exponential curves of the general form:

y = *k* × e^−*at*^ (3)

in which *k* and *a* are constants and *t* is the reading time of the anti-elastase activity. Considering this, the choice adopted to study the data was to use principal component analysis (PCA) to examine the internal correlation structure of the dataset and verify in what relationship the different curves of the anti-elastase readings were. The data were then autoscaled and the study of the relationships between the variables S, C and M and the reading times of the anti-elastase activity was conducted using the PCA directly on the data without further preprocessing.

### 2.6. Optimization and Validation of the Manufacturing Process

Following the information collected by analyzing protein and lipid yields and in vitro anti-elastase activity, which revealed substantial batch-to-batch variability, the process has been optimized. Briefly, all the procedures described in Section 2.3 have been repeated, but the concentration step was stopped when a protein concentration of 115 µg/mL was reached (the value was conventionally selected). In addition, characterization in terms of protein, lipid content and anti-elastase activity has been performed on the new batches prepared according to the procedures reported in Section 2.4.3, Section 2.4.4 and Section 2.4.6. The control charts for protein and lipid content have been designed and the anti-elastase activity data have been analyzed by the PCA analysis as described in Section 2.5.

## 3. Results and Discussion

Many challenges still need to be faced in the transition of MSC-secretome therapies into the clinic. These include practical problems, such as the need for an adequate number of cells (which may be solved using bioreactors [25]), the necessity of scalable, reproducible and GMP-compliant manufacturing protocols and the MSC-secretome formulation into a standardized pharmaceutical dosage form (possibly in a steady dry state). In this regard, starting from a method that combines ultrafiltration and freeze-drying to transform MSC-secretome into a pharmaceutical product–the lyosecretome, this work used a multi-level 5 × 2 experimental design to develop a freeze-dried formulation of MSC-secretome by using a mannitol/sucrose binary mixture.

**Figure 1:**
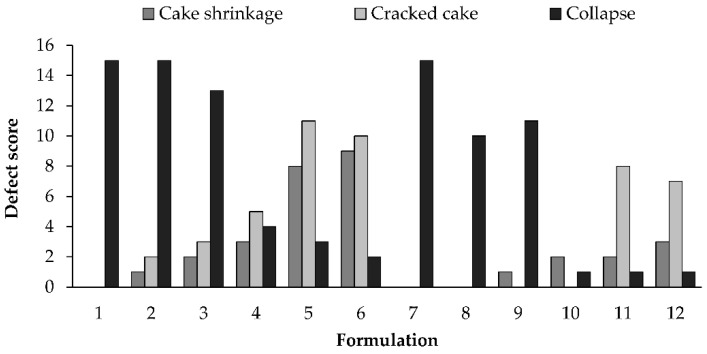
Defect scores for the evaluated formulations.

At first, the residual humidity and the cake aspect have been evaluated. Residual moisture did not exceed 4%, indicating an effective drying of the products and that the different excipients ratio does not affect this parameter. Three primary defects were noted in cake appearance evaluation: collapse, cake shrinkage and cracked cake (Figure S1). Figure 1 represents the defect scores plotted as a function of the different formulations. For the formulations containing a low amount of mannitol (i.e., the high amount of sucrose), the only defect observed was the collapse of the cake. The collapse score decreased by increasing the amount of mannitol and an increase of cracks in the cake was observed. Even the defect “Cake Shrinkage” increased by increasing the mannitol amount. These findings can be explained by the ability of mannitol to crystallize and by the behaviour of sucrose, which produces amorphous structures. The increase of the amount of mannitol in the formulation reduces the cakes collapse, leading the evaluators to notice other defects that are not masked or do not occur when the collapse is present. These defects (i.e., skin formation, minor splashing and non-uniform cake appearance) were not reported because they were rarely noticed.

Then, the protein and lipid yield, the EV particle size and the in vitro anti-elastase activity of the prepared samples were studied by evaluating the effect of the secretome batch (S), the lyophilization (L), the concentration of excipients (C) and the concentration ratio of mannitol and sucrose, as detailed below. 

### 3.1. Model for the “Protein Content” Response, Y_P_

The data acquired on the protein yield (Table S2) are not conclusive. As shown in Figure S2, the experimental variance of the results is too high and the replicated data show to be aggregated “in series”. The predictive ability of the model, therefore, is not good. In some cases, the estimate predicted by the model is very far from the observed value (Table S3 and Figure S3). However, only considering the trend indicated by the data through the calculated model, two effects are evident: (i) the first, more important, is due to the secretome preparation batch and (ii) the second derives from freeze-drying. For the first effect, we noted that batch 1 has an amount of protein higher than batch 2 and 3, while for the second effect, it was noticed that the freeze-drying process reduces the amount of protein (the protein amount is greater before freeze-drying). This aspect can be explained considering that the stresses generated during the freeze-drying process can alter the reducing power of proteins (thus, the BCA kit detect a low amount of proteins because less Cu^2+^ is reduced to Cu^+^). The factors C, the concentration of excipients and M, the proportion of mannitol in the preparation, on the other hand, do not influence the Y_P_ response. Likely, each mixture of excipients selected can preserve the protein content in the range of concentrations considered. However, it was not possible to increase the total amount of the excipients because the product would be exceedingly diluted to have biological activity. Moreover, at high concentrations of excipients, safety is compromised after administration due to hyperosmolarity. The model equation is:

Y_P_ = 24.83 + 165.78 S1^(^***^)^ − 12.24 S_2_ − 28.18 L^(^***^)^ − 2.59 C − 0.81 M − 2.00 C M + 0.48 M^2^ (4)

*** means *p* < 0.001.

**Figure 2:**
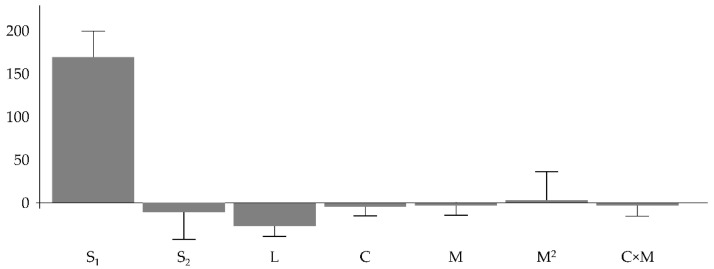
Coefficients for the model Y_P_.

Figure 2 shows the magnitude and sign of the coefficients. Their statistical significance is meaningless since the model is not validated (see Table S3 and Figures S2 and S3).

**Figure 3:**
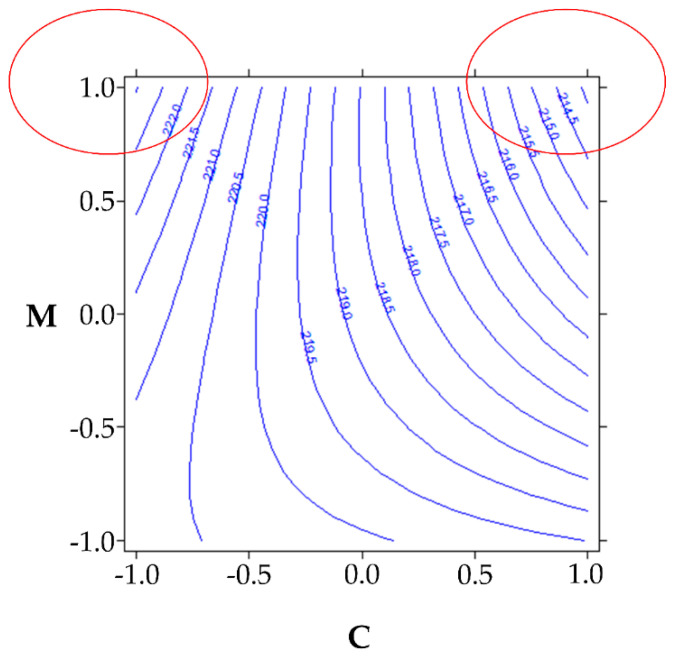
Isoresponse curves plot for batch 1 before freeze-drying.

In batch 1, before freeze-drying, the maximum protein content is achieved when the maximum amount of mannitol is used, regardless of the total amount of excipients (Figure 3). This is probably because mannitol is more active than sucrose in the protection of proteins. Therefore, when the mannitol proportion increases, this effect is expected to be greater. If, on the other hand, C (which is given by mannitol + sucrose) increases, the effect of the less effective (sucrose) predominates and therefore interacts with the effect provided by mannitol.

### 3.2. Model for the “Lipid Content” Response, Y_L_

**Figure 4:**
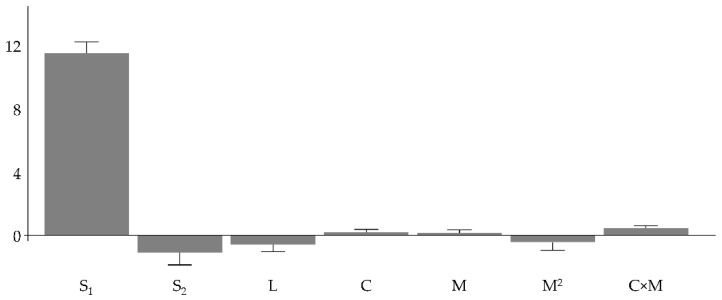
Coefficients for the model Y_L_.

The observations illustrated for the model relating to the “protein content” response (Y_P_), were replicated almost identical in the “lipid content” response, Y_L_. The critical difference between the two models is that here the model for the Y_L_ response is validated and can be considered valid for describing the trend of the results (see Table S4). So, in the case of the lipid content, the two effects described above, i.e., the effects of batch production and lyophilization, are evidenced. As shown in Figure 4 and by the model Equation (5), the first and the most relevant effect on the response is the secretome batch. Batch 1 shows the greater lipid content, whereas batch 2 has the lowest lipid content.

Lyophilization decreases the lipid content. This aspect can be explained considering that the stresses generated during the freeze-drying process can damage the lipidic layer (thus, the Nile fluorescence is lowered). Factors C, the excipients’ concentration and M, mannitol proportion in the preparation are also in this case almost irrelevant to the response. The model equation is:

Y_L_ = 1.84 + 11.59·S_1_ ^(^***^)^ − 0.91·S_2_^(^*^)^ − 0.58·L^(^**^)^ + 0.15·C + 0.06·M + 0.28·C·M − 0.34·M^2^ (5)

* means *p* < 0.05; ** means *p* < 0.01; *** means *p* < 0.001.

Figure 4 shows the coefficients magnitude and sign together with the confidence interval of their mean value. The coefficients of the factors C and M are not statistically significant and are numerically negligible compared to the coefficient of the batch effect (S_1_).

Overall, the data document an important batch effect for both the “protein content”, Y_P_ and “lipid content”, Y_L_ responses. The conditions that provide the preparations with both responses largest numerically are batch 1 and no lyophilization. The total concentration of excipients (C) and the ratio between the two sugars do not influence the two responses. The data examination also indicates that if the batch effect could be eliminated, the two responses would be maximum in the condition of before lyophilization, with the maximum total concentration of excipients (1.5% *w*/*v*) and with an M:S ratio of about 60% *w*/*w*. Accordingly, it was hypothesized that the maximum total concentration of excipients and an M:S ratio of about 60% *w*/*w* better preserve proteins and lipids during the lyophilization process.

### 3.3. Model for the “EV Particle Size” Response, Y_NTA_

The data presented in Table S5 about the mode, the first decile (d_10_) and the ninth decile (d_90_) of freeze-dried EVs are not described by the postulated model reported above (see Equation (2)). In particular, the value of the coefficient of determination adjusted for the degrees of freedom (R^2^_adj_) is equal to −0.059 for the mode, −0.055 for d_10_ and 0.230 for d_90_. This result is to be interpreted assuming that none of the factors considered (S, C and M) affects the EV particle sizes in the prepared freeze-dried secretomes.

### 3.4. Model for the “In Vitro Anti-Elastase Activity” Response, Y_A_

**Figure 5:**
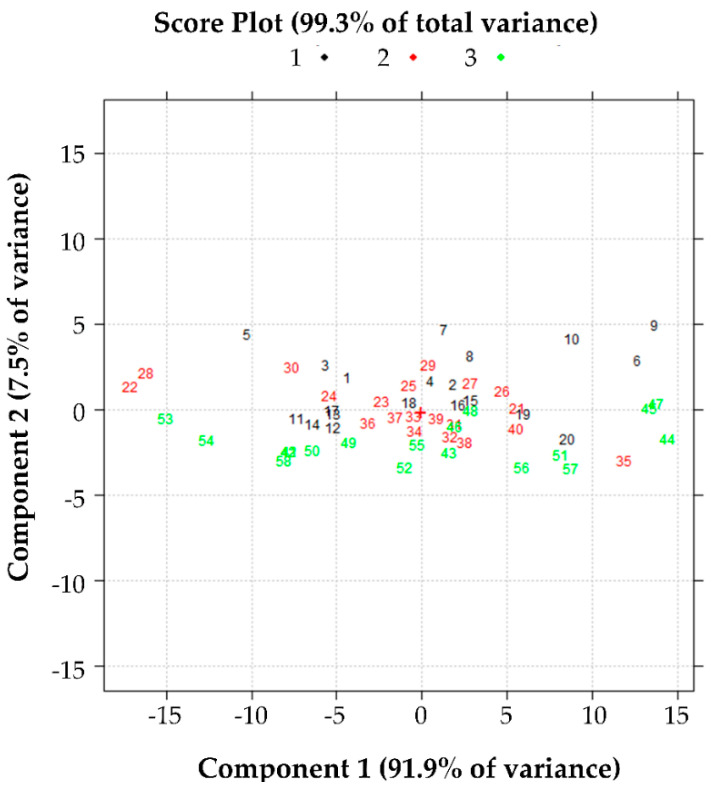
Effect of variable S, the batch production, on the anti-elastase activity. Score plot in the plane of the first two principal components of the data reported in Table S1.

Using the PCA, the relationships between S, C and M and the anti-elastase activity values were investigated. The graph of Figure 5 shows that the data are described in the plane of the first two principal components (PC1 and PC2) with an explained variance equal to 99.3%. This indicates that almost all the variance of the results collected is explained in this plane. Furthermore, the color codes show a secretome batch effect in the anti-elastase activity to discriminate batch 3 (green points) from batches 1 and 2 (red and black points, respectively). However, the discrimination is evident only along the PC2 and corresponds to an explained variance of 7.5%.

**Figure 6:**
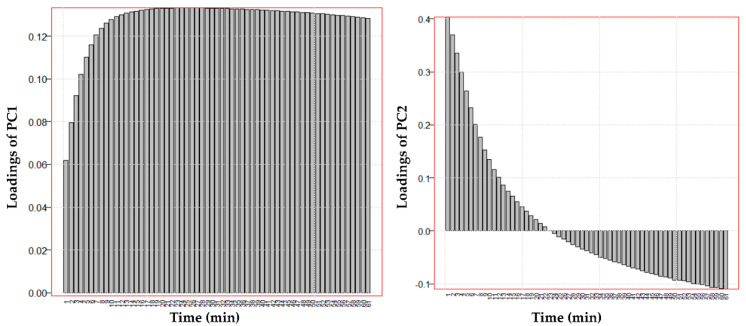
Loadings of the two principal components of the data reported in Table S1 as a function of time.

**Figure 7:**
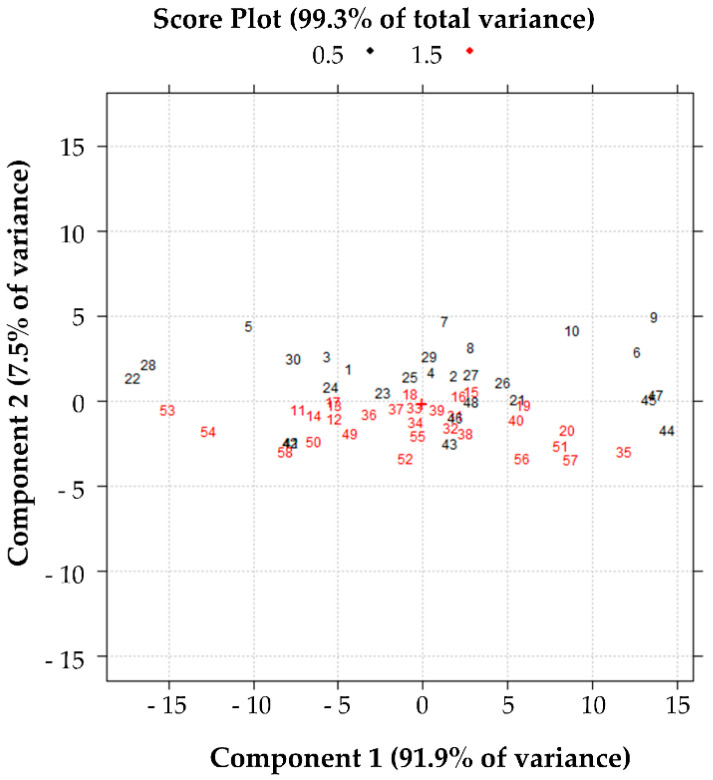
Effect of variable C on the anti-elastase activity. Score plot in the plane of the first two principal components of the data reported in Table S1.

**Figure 8:**
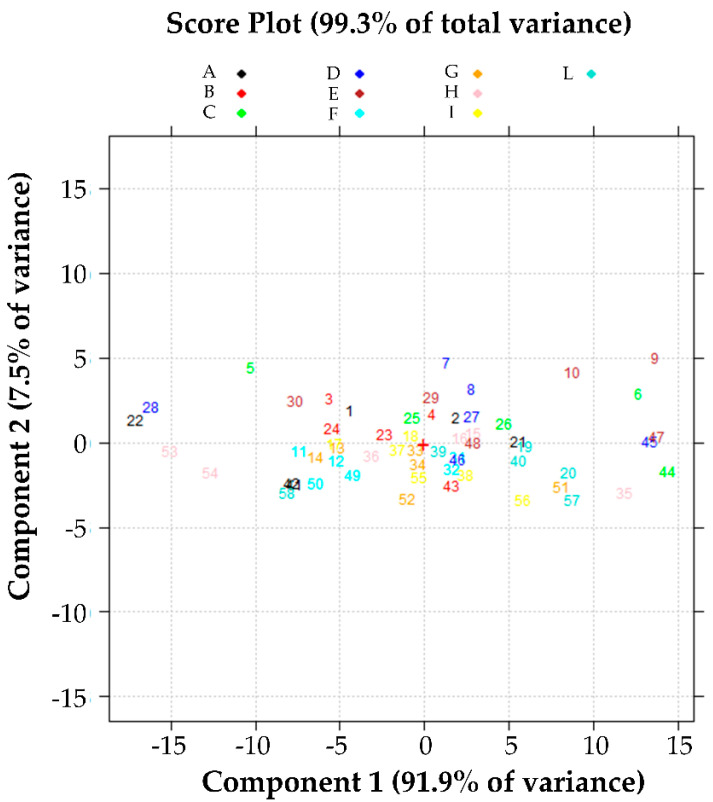
Effect of the variable M on the anti-elastase activity. Score plot in the plane of the first two principal components of the data reported in Table S1.

Along the PC1, the discrimination between the batches is not evident. However, by observing the loadings values in the bar graph of Figure 6, it can be better understood what is explained by the PC1. Indeed, PC1 reads the variation in anti-elastase activity from about 10 min to 61 min with positive loadings values. Therefore, on PC1 it is represented how the anti-elastase activity grows over time for each sample studied as a function of the three variables examined (S, C and M). As it was already clear from the data, the anti-elastase activity increases exponentially from the beginning of the experiment and reaches a maximum but apparently asymptotic value at the end of the experiment. PC1 then shows the samples with the higher anti-elastase activity, especially from about the tenth minute of reading until the end of the experiment. Thus, higher values of anti-elastase activity correspond to higher values of PC1 in this time interval. The loadings on PC2 show how the anti-elastase activity reading varies in the first minutes of the experiment: positive loadings are computed from about 1 min onwards up to about 20 min and negative from 25 min at the end of the experiment. These observations help to understand the role of variables C and M, illustrated in subsequent Figure 7 and Figure 8, respectively.

The data illustrated in Figure 7 show that the samples numbered in black have anti-elastase activity greater at the beginning of the experiment (from 1 to about 20 min). In comparison, those numbered in red have a greater variation in anti-elastase activity at the end of the experiment (from 25 min onwards). In general, the samples with greater anti-elastase activity at shorter read times are the rows n. 6, 9, 10 (batch 1), 44, 45 and 47 in black (batch 3) with C at level 0.5% *w*/*v*. The samples with greater anti-elastase activity at longer read times and with C at the level of 1.5% *w*/*v* are those of rows n. 20 (batch 1), 35 (batch 2), 51 and 57 (batch 3) (refer to Table S1).

The picture of the results represented in Figure 8, on the other hand, is less clear but shows the correspondence of the values of the anti-elastase activity as a function of M.

In conclusion, the anti-elastase activity depends on (i) the secretome batch: the anti-elastase activity of batch 3 is different from that of batches 1 and 2 (Figure 5). (ii) by the factor C; samples have anti-elastase activity dependent on factor C level and time. When C is at level 0.5% *w*/*v*, the highest anti-elastase activity is observed in the first minutes of the experiment (from 1 to 15 min approximately). From 15 min onwards, samples with C at the level of 1.5% *w*/*v* have higher anti-elastase activity. (iii) The effect of M as such, is unclear and attributable to the two effects already described. It is essential to note that factors C and M were evaluated, as reported in Section 3.1 and Section 3.2, quantitatively and not qualitatively. Likely, different C and M values better preserve different MSC-secretome components, which influence the anti-elastase activity. Overall, based on such results, we concluded that the optimal formulation is the one already reported with 0.5% *w*/*v* mannitol, which showed to be active also in vivo [26].

### 3.5. Optimization and Validation of the Manufacturing Process

**Figure 9:**
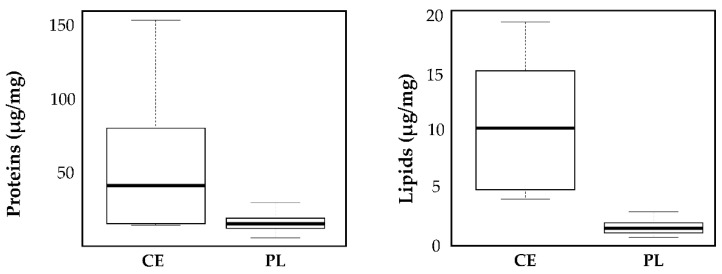
Results relative to the assays of proteins and lipids. CE = standardization for cell equivalents; PL = protein/lipid standardization.

The data analysis reported in Section 3.1, Section 3.2, Section 3.3, Section 3.4 revealed that the batch strongly influences the product performances (protein and lipid yields and in vitro anti-elastase activity). Therefore, the ultrafiltration process used to isolate MSC-secretome from cell culture supernatants, which involves the standardization of the final product in cell equivalents (CE), should be improved. In this regard, the process has been modified so that the final product is standardized to a specific protein concentration (conventionally fixed at 115 µg/mL, PL) instead of CE. It has to be noted that, as a consequence, also the lipid content was standardized (the mean ± S.D. lipid content is 1.62 ± 0.71), probably because proteins and lipids have the same ratio in MSC-secretome. Therefore, the data of proteins and lipids (Table S6) were studied to verify the effect of PL or CE standardization. Figure 9 shows the box plots of the data as they are.

**Figure 10:**
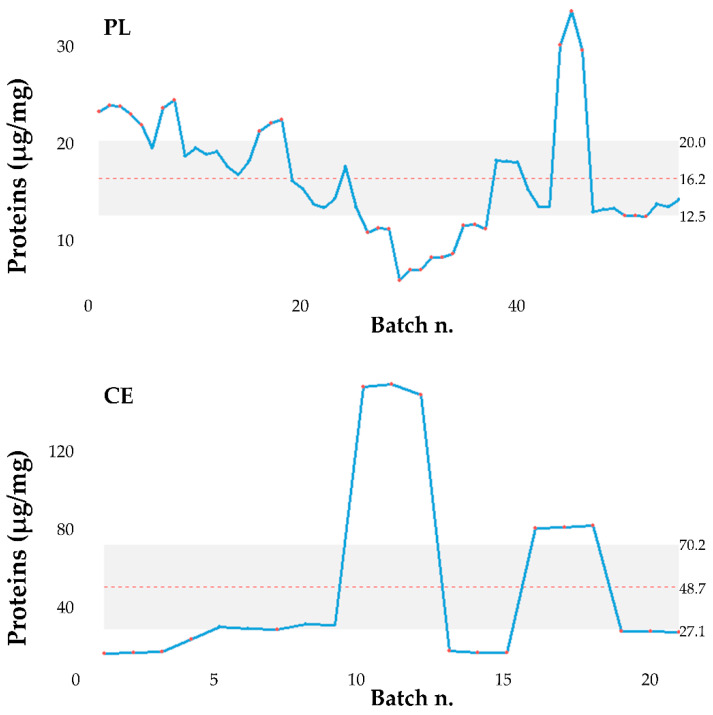
Control charts of the protein content in the batches studied. CE = standardization for cell equivalents; PL = protein/lipid standardization.

**Figure 11:**
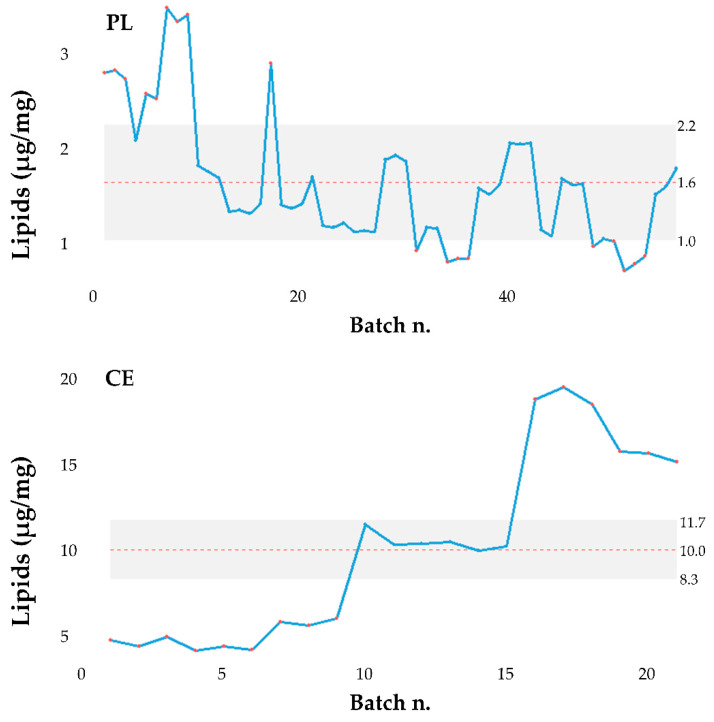
Control charts of the lipid content in the batches studied. CE = standardization for cell equivalents; PL = protein/lipid standardization.

The two groups of data, about both protein and lipid dosages, are heteroskedastic and therefore do not satisfy one of the basic hypotheses that allow the use of ANOVA for data comparison. However, the comparison with hypothesis tests (which should necessarily be non-parametric) is entirely useless since, as expected, it is clear that the data are much more precise if the standardization is carried out with the PL method. In both cases, the median (the bold line) of the data measurements standardized with the PL method is lower than that obtained with CE standardization. The control charts of the same data are shown in Figure 10 and Figure 11 below.

**Figure 12:**
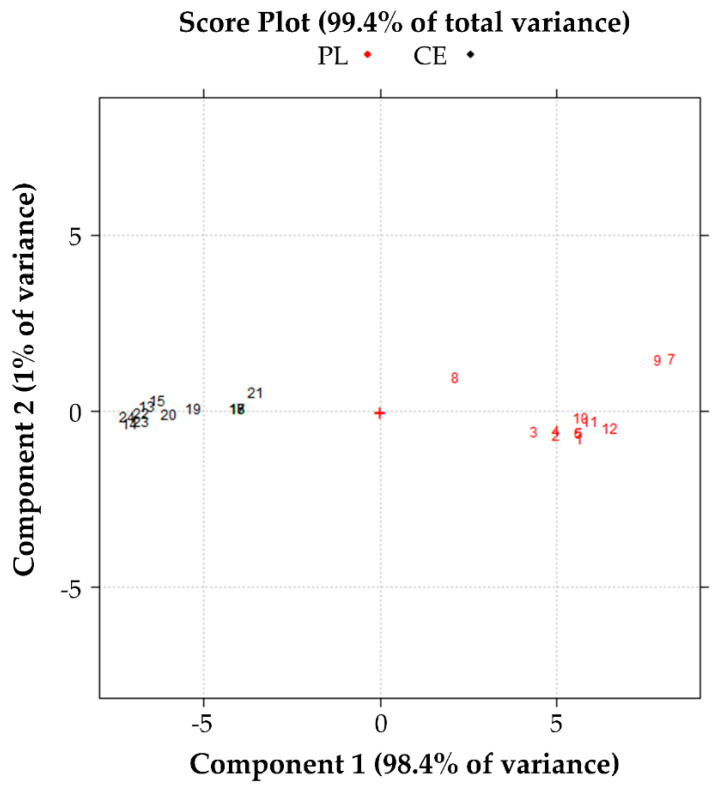
Effect of the batch on the anti-elastase activity. Score plot in the plane of the first two principal components of the data reported in Table S7.

Finally, the data of anti-elastase activity (Table S7) were studied by PCA. The score plot shown in Figure 12 shows that the data are well described in the plane of the first two PCs, with an explained variance of 99.4%. There is evident discrimination of the samples along with PC1, while the PC2 now explains only about 1% of the total variance of the data.

**Figure 13:**
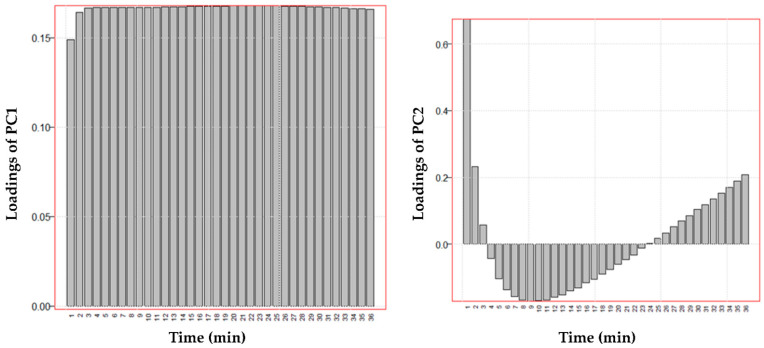
Loadings of the first two principal components of the data reported in Table S7 as a function of time.

Standardization with the PL method (red dots) produces batches with anti-elastase activity higher than that highlighted by the batches standardized with the CE method (black dots). In this case, PC1 describes the variation of the anti-elastase activity for almost all of the reading experiments. In contrast, the PC2 describes the variation of the residual anti-elastase activity in the first 2 min of the test (Figure 13). Therefore, almost all of the information provided by the anti-elastase activity readings is summarized in PC1 (Figure 13).

## 4. Conclusions

Producing MSC-secretome for dose escalation studies and clinical practice requires scalable and GMP-compliant production procedures and formulation into a standardized medicinal product. Starting from a method that combines ultrafiltration and freeze-drying to transform MSC-secretome into a pharmaceutical product—the lyosecretome, in this work, a multi-level 5 × 2 experimental design was used to study the influence of mannitol(M)/sucrose(S) binary mixture, selected as an excipient. A quadratic D-optimal design was computed to evaluate the effect of secretome batch (S), lyophilization (L), the concentration of excipients (C) and the mannitol/sucrose ratio in the excipient mixture (M) on the pharmaceutical dosage form characteristics (residual humidity and cake appearance) and on the product performances (protein and lipid yields, the EV particle size and in vitro anti-elastase activity). The different excipients ratio did not affect residual moisture or EV particle size; simultaneously, proteins and lipids were better preserved in the freeze-dried product using the maximum total concentration of excipients (1.5% *w*/*v*) with an M:S ratio of around 60% *w*/*w*. On the other hand, the anti-elastase activity was better preserved using 0.5% *w*/*w* of mannitol as an excipient. The secretome batch resulted in being the primary source of variability; therefore, the manufacturing process has been modified and validated: the final product is now concentrated to reach a specific protein (and lipid) concentration instead of cell equivalents. This change in the quality assessment of our product resulted in a robust process and a tentative set of quality attributes that lead to a successful scale-up of the process.

Overall, this manuscript suggests a strategy to optimize the preparation and formulation of MSC-secretome, which can be applied, with suitable modifications, even to other biological drugs. Indeed, as the product is the process, any slight change in the production process can modify the qualitative-quantitative composition of the finished product and, therefore, its biological activity. Thus, through the concept of quality by design, the quality of the pharmaceutical product has to be built from the beginning, by complementary characterization techniques (validated at pharmaceutical grade and with an acceptable cost to be implemented on the quality controls that are carried out routinely) and by evaluating the expected therapeutic effect using standardized potency assays that possibly reflect the hypothesized MoA. In this regard, we chose the anti-elastase activity as we previously demonstrated that it is linked to the presence of elastase inhibitors, like alpha 1 antitrypsin [14].

## Figures and Tables

**Table 1:** Details of the factorial design used.

Formulation	V1	V2	V1 = Mannitol %	% *w*/*v*
1	−1.0	−1.0	25	0.5
2	−0.5	−1.0	45	0.5
3	0.0	−1.0	65	0.5
4	0.6	−1.0	85	0.5
5	1.0	−1.0	100	0.5
6	−1.0	+1.0	25	1.5
7	−0.5	+1.0	45	1.5
8	0.0	+1.0	65	1.5
9	0.6	+1.0	85	1.5
10	1.0	+1.0	100	1.5

**Table 2:** Parameters of the lyophilization process.

Phase	Section	Time (h:min)	Shelf’s Temperature (°C)	Pressure (mbar)
Loading	1	-	25	1000
Freezing	2-ramp	0:20	5	
	3-hold	0:40	5	
	4-ramp	0:10	−5	
	5-hold	1:00	−5	
	6-ramp	0:45	−45	
	7-hold	4:30	−45	
Primary drying	8	0:20	−45	0.08
	9	0:30	−40	
	10	79:09	−40	
Secondary drying	11	2:30	22	
	12	8:00	22	

**Table 3:** Factors and levels studied.

Factors	Levels	Encoding
Secretome batch (S)	1	S_1_ = 1, S_2_ = 0
	2	S_1_ = 0, S_2_ = 1
	3	S_1_ = S_2_ = 0
Lyophilization (L)	Pre	1
	Post	+1
Total concentration of excipients (% *w*/*v*)	0.5	−1
	1.5	+1
Mannitol/sucrose concentration ratio (% *w*/*w*)	25/75	−1
	100/0	+1

## Data Availability

The data presented in this study are contained within the article and its Supplementary Materials.

## Supplementary Materials

The following are available online at https://www.mdpi.com/article/10.3390/pharmaceutics13081129/s1, Figure S1. Cake aspect of freeze-dried Lyosecretome (numbers identify the formulation as detailed in Table 1). Figure S2. The plot of residuals for the model YP. Figure S3. Fitting for the model YP. Table S1. Summary of the in vitro elastase activity data; Table S2. Summary of the protein and lipid content. Table S3. The predictive ability for the model Y_P_. NA = Not applicable. Table S4. The predictive ability for the model Y_L_. NA = Not applicable. Table S5. Experimental plan (data in black), experimental matrix (in blue) and responses (in the green field) were studied for the analysis of the data of the diameter of the lyophilized particles. Table S6. Summary of the protein and lipid content for batches standardized in proteins/lipids (PL) or cell equivalents (CE). Table S7. Summary of the anti-elastase data for batches standardized in proteins/lipids (PL) or cell equivalents (CE).
