# Supplementary material for: Freeze-Dried Mesenchymal Stem Cell-Secretome Pharmaceuticalization: Optimization of Formulation and Manufacturing Process Robustness
Source: Pharmaceutics. 2021 Jul 23;13(8):1129. doi: 10.3390/pharmaceutics13081129 (PMC8401234; doi:10.3390/pharmaceutics13081129)
Supplement: Supplementary file 1 [file pharmaceutics-13-01129-s001.zip › pharmaceutics-1291685-supplementary/supplementary material/pharmaceutics-1291685-supplementary.pdf]

pharmaceutics

Supplementary material: Freeze-Dried Mesenchymal Stem Cell-Secretome Pharmaceuticalization: Optimization of Formulation and Manufacturing Process Robustness

Michela Mocchi, Elia Bari, Giorgio Marrubini, Andrea Foglio Bonda, Sara Perteghella, Fulvio Tartara, Fabio Cofano, Giuseppe di Perna, Lorella Giovannelli, Delia Mandracchia, Marzio Sorlini, Diego Garbossa, Maria Luisa Torre and Lorena Segale

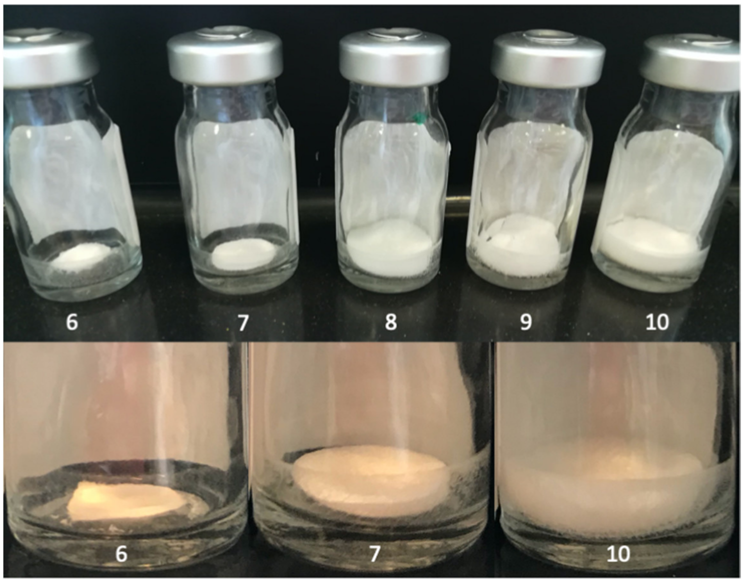

Figure S1. Cake aspect of freeze-dried Lyosecretome (numbers identify the formulation as detailed in Table 1).

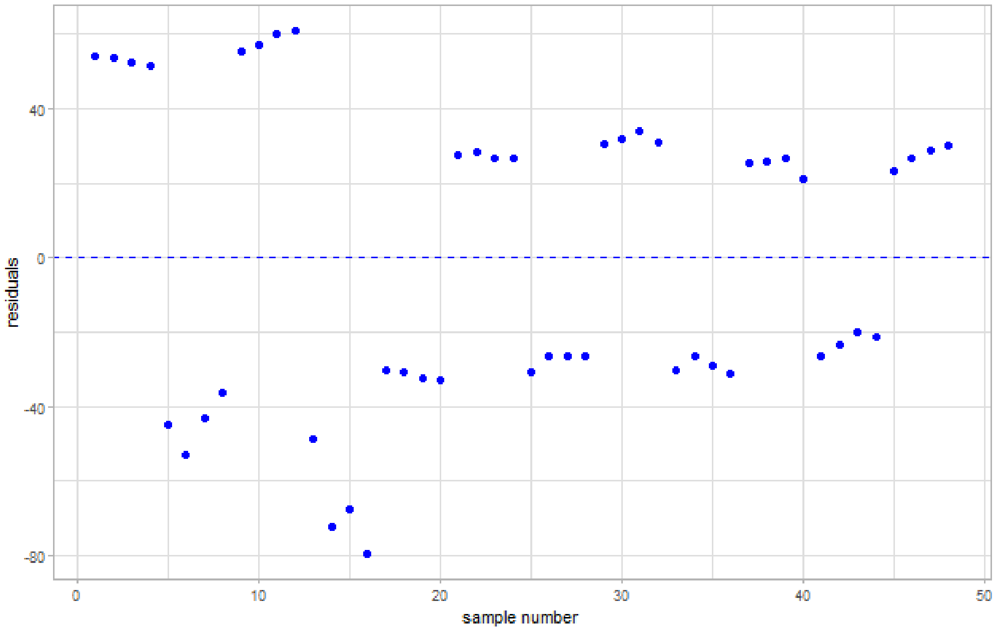

Figure S2. The plot of residuals for the model Y_P.

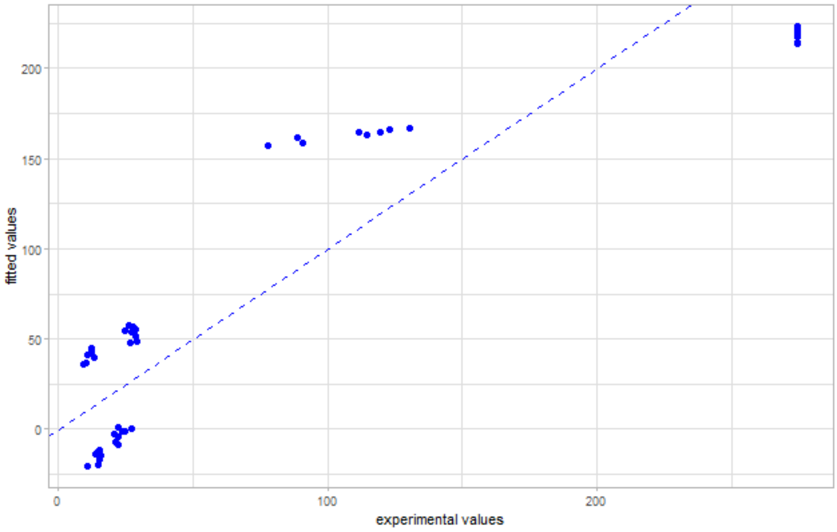

Figure S3. Fitting for the model Y_P.

Table S3. The predictive ability for the model Y_P. NA = Not applicable.

Test	Secretome batch	Lyophilization	Concentration	Mannitol (%)	Experimental Y_P	Calculated Y_P	Relative error %
4	1	Pre	0.5	65	274.76	221.46	19
10	1	Post	0.5	65	116.48	165.11	−42
16	1	Pre	1.5	65	274.76	216.02	21
22	1	Post	1.5	65	92.23	159.66	−73
4	2	Pre	0.5	65	13.19	43.45	−229
10	2	Post	0.5	65	14.89	−12.91	187
16	2	Pre	1.5	65	12.61	38.00	−201
22	2	Post	1.5	65	14.58	−18.35	226
4	3	Pre	0.5	65	25.93	55.68	−115
10	3	Post	0.5	65	23.28	−0.67	NA
16	3	Pre	1.5	65	27.09	50.24	−85
22	3	Post	1.5	65	21.05	−6.12	129

Table S4. The predictive ability for the model Y_L. NA = Not applicable.

Test	Secretome batch	Lyophilization	Concentration	Mannitol (%)	Experimental Y_L	Calculated Y_L	Relative error %
4	1	Pre	0.5	65	14.56	13.85	5
10	1	Post	0.5	65	10.32	12.72	−23
16	1	Pre	1.5	65	14.56	14.18	3
22	1	Post	1.5	65	14.49	12.98	10
4	2	Pre	0.5	65	1.20	1.34	−12
10	2	Post	0.5	65	0.00	0.22	100
16	2	Pre	1.5	65	0.73	1.68	−130
22	2	Post	1.5	65	0.48	0.48	−1
4	3	Pre	0.5	65	1.92	2.25	−17
10	3	Post	0.5	65	1.87	1.13	40
16	3	Pre	1.5	65	1.68	2.59	−54
22	3	Post	1.5	65	1.15	1.41	−23

Table S5. Experimental plan (data in black), experimental matrix (in blue) and responses (in green field) studied for the analysis of the data of the diameter of the lyophilizate particles.

Test	Secretome batch	C	M	S1	S2	C	M	Mode	d_{10}	d_{90}
8	1	0.5	25	1	0	-1	-1.0	92.07	74.83	199
9	1	0.5	45	1	0	-1	-0.5	117	105.6	294
10	1	0.5	65	1	0	-1	0.0	97.1	89.4	195.2
11	1	0.5	85	1	0	-1	0.6	93.20	86.2	263.3
12	1	0.5	100	1	0	-1	1.0	98.50	92.5	341.2
20	1	1.5	25	1	0	1	-1.0	88.1	79.3	238
21	1	1.5	45	1	0	1	-0.5	96.3	85.9	240.6
22	1	1.5	65	1	0	1	0.0	96.5	84.5	259.4
23	1	1.5	85	1	0	1	0.6	130.5	124.4	359.3
24	1	1.5	100	1	0	1	1.0	153.8	129.5	487
8	2	0.5	25	0	1	-1	-1.0	110.6	96.55	266.05
9	2	0.5	45	0	1	-1	-0.5	102.2	98.4	280
10	2	0.5	65	0	1	-1	0.0	105.5	82.6	219.1
11	2	0.5	85	0	1	-1	0.6	84.90	81.4	232.5
12	2	0.5	100	0	1	-1	1.0	100.20	89.2	326.6
20	2	1.5	25	0	1	1	-1.0	87.3	83.4	269.9
21	2	1.5	45	0	1	1	-0.5	96.4	91.1	274.4
22	2	1.5	65	0	1	1	0.0	75.8	71.2	174.5
23	2	1.5	85	0	1	1	0.6	126.9	82.4	189.6
24	2	1.5	100	0	1	1	1.0	93.4	85.7	231.1
8	3	0.5	25	0	0	-1	-1.0	108.9	102	316.3
9	3	0.5	45	0	0	-1	-0.5	119.6	108.8	248
10	3	0.5	65	0	0	-1	0.0	75.8	65.55	166.65
11	3	0.5	85	0	0	-1	0.6	91.50	86.2	276.8
12	3	0.5	100	0	0	-1	1.0	95.10	87.2	286.1
20	3	1.5	25	0	0	1	-1.0	100.3	89.4	328.1
21	3	1.5	45	0	0	1	-0.5	102.1	89.7	254.3
22	3	1.5	65	0	0	1	0.0	96.2	85	246.4
23	3	1.5	85	0	0	1	0.6	112.4	101.1	258.6
24	3	1.5	100	0	0	1	1.0	106.8	90	281.9
